# Cardiac Transthyretin Amyloidosis: Hidden in Plain Sight

**DOI:** 10.1155/2021/2551964

**Published:** 2021-12-07

**Authors:** Constantine N. Logothetis, Joel Fernandez, Damian A. Laber

**Affiliations:** ^1^Department of Internal Medicine, Morsani College of Medicine, University of South Florida, Tampa, FL, USA; ^2^Division of Cardiovascular Medicine, Morsani College of Medicine, University of South Florida, Tampa, FL, USA; ^3^Section of Satellite and Community Oncology, H. Lee Moffitt Cancer Center and Research Institute, Tampa, FL, USA

## Abstract

Amyloidosis is an underappreciated medical condition with symptoms camouflaging as common medical comorbidities leading to its underdiagnosis due to its systemic involvement. Despite common misconceptions, amyloidosis and its systemic comorbidities are more prevalent and treatable than previously acknowledged by the medical community. There are two major forms of amyloidosis: amyloid light-chain and transthyretin amyloidosis. Each of these have a distinct pathophysiology, diagnostic work-up, treatment, and prognosis. The patient described in this study was diagnosed with transthyretin cardiac amyloidosis months after presenting with heart failure of unknown etiology. Usually, clinicians presume that heart failure results from common comorbidities such as hypertension, diabetes, and hyperlipidemia. Here, the correct etiology was transthyretin cardiac amyloidosis. The patient had five admissions for heart failure symptoms prior to a physician identifying the etiology as cardiac transthyretin amyloidosis. After initiating the transthyretin stabilizer tafamidis, the patient did not experience another heart failure exacerbation. This vignette provides an example of the clinical presentation, diagnostic work-up, and treatment of a patient with cardiac transthyretin amyloidosis. The review of the literature focuses on the epidemiology, and clinical symptoms that should prompt an evaluation for cardiac amyloidosis as well as the diagnostic and therapeutic options are available. Transthyretin cardiac amyloidosis is a rare and underdiagnosed disease, while heart failure is a highly prevalent condition. This clinical vignette seeks to provide education and awareness to an overlooked medical disorder.

## 1. Introduction

Amyloidosis is a heterogeneous illness with varying pathophysiology and a spectrum of clinical presentation due to its systemic involvement. There are two well defined pathophysiologies of amyloidosis, amyloid light-chains (AL) amyloidosis, and amyloid transthyretin (ATTR) amyloidosis, and their mechanism of disease is radically different. ATTR amyloidosis can be further subcategorized into variant ATTR (ATTRV) or wild-type ATTR (ATTRWT). One complication of this condition is cardiac amyloidosis leading to congestive heart failure (CHF). CHF from ATTR is often overlooked for more common causes of CHF such as hypertension or diabetes. Accurate diagnosis is imperative as appropriate treatment, and prognostication is dictated by the type of cardiac amyloidosis. This clinical vignette and review of the literature provides a brief overview of amyloidosis, while focusing on the difficulties in diagnosing transthyretin cardiac amyloidosis, and the high index of suspicion required to detect these patients.

## 2. Case Presentation

A 75-year-old African American (AA) male with recently diagnosed congestive heart failure (CHF) presented to our hospital with shortness of breath, persistent cough, occasional hemoptysis, abdominal fullness, lower extremity swelling, and an eight-pound weight gain over a week. These symptoms had been progressively worsening for six months. He was admitted to the cardiac intensive care unit for further management.

Three months prior to this presentation, he was hospitalized at another facility with similar symptoms and diagnosed with CHF. He underwent myocardial perfusion imaging which was normal and a transthoracic echo (TTE) that reported severe left ventricular hypertrophy (LVH) and an ejection fraction (EF) of 60–65%. At that time, brain-natriuretic peptide (BNP) was 425 pg/mL. He improved with standard CHF therapy, including furosemide, carvedilol, and losartan. After that, he had three additional admissions for similar symptoms. The medical history included hypertension, noninsulin-dependent type II diabetes mellitus, hyperlipidemia, chronic obstructive pulmonary disease, chronic kidney disease stage III, and obstructive sleep apnea. His family history included cardiomyopathy in his father and brother; however, the patient was unaware of the specific details regarding the type of cardiomyopathy. His examination demonstrated bibasilar crackles in the lungs, bilateral 2+ pitting edema in the lower extremities, and jugular venous distension up to the earlobe sitting at a 45° angle. Vital signs showed heart rate 76 beats per minutes, respiratory rate of 18 per minute, blood pressure 144/72 mmHg, and temperature 36.7°C.

**Figure 1:**
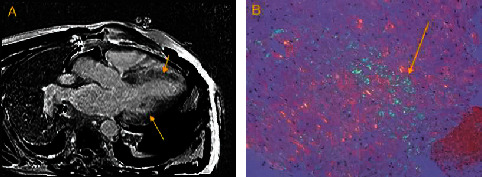
(a) Four-chamber heart view on cardiac MRI with diffuse late gadolinium enhancement (DLGE) (yellow arrows). DLGE occurs as a consequence of regional differences in the myocardial extracellular volume and can be an indicator of an infiltrative process. (b) Tissue from an endomyocardial biopsy with apple-green birefringence under polarized light with Congo red stain (yellow arrow).

Laboratory tests revealed hemoglobin 10.2 g/dL, creatinine 1.8 mg/dL (baseline 1.6–1.8), troponin 0.169 ng/dL, and BNP 340 pg/mL. Electrocardiogram (ECG) revealed sinus rhythm with first degree atrioventricular block, left atrial enlargement, and evidence of a prior infarct with Q-waves in V1-V2 and nonspecific T-waves in leads I and aVL. Thyroid stimulating hormone was normal. Troponins did not peak after three consecutive values. A TTE demonstrated severe LVH, EF of 55–60% with no regional wall abnormalities, and Doppler pattern was consistent with a restrictive physiology. Given the findings of restrictive physiology on TTE, a cardiac magnetic resonance imaging (MRI) was done. The cardiac MRI revealed an EF of 63% by Simpson's method, normal systolic function, no regional wall abnormalities, and severe basal and midseptal hypertrophy. There was patchy, diffuse late gadolinium enhancement (DLGE) in the myocardium (Figure 1(a)). A right and left heart catheterization (LHC) with endomyocardial biopsy (EMB) was performed to thoroughly assess his hemodynamics, image his coronary arteries, and evaluate for amyloidosis. Hemodynamics revealed moderate group II pulmonary hypertension with a normal cardiac output by Fick calculations. His pulmonary capillary wedge pressure was 31 mmHg. LHC demonstrated nonobstructive coronary artery disease. He was treated with aspirin 325 mg carvedilol, furosemide, and hydralazine with improvement in his symptoms and was discharged home.

The tissue pathology from EMB revealed apple-green birefringence under polarized light with Congo red stain consistent with amyloid deposition (Figure 1(b)). Immunoelectrophoresis of the blood and urine and flow cytometry of peripheral blood leucocytes were all within normal limits. Genetic testing demonstrated a heterozygous TTR-V142I mutation consistent with hereditary ATTR amyloidosis. He started tafamidis with continued improvement and no more CHF exacerbations or hospitalizations. Given his neuropathy, he has been evaluated by neurology for treatment with patisiran for amyloidosis-induced neuropathy.

## 3. Discussion

ATTR amyloidosis is a rare disease that has an increase in all-cause mortality and cardiovascular-related hospitalizations if undiagnosed and untreated. ATTRwt is a rare disorder usually diagnosed in the 7^th^–10^th^ decade of life and ATTRv being diagnosed in the 3^rd^–8^th^ decade of life. Males are principally affected by both types of ATTR; however, 90% of patients with ATTRwt are male [1]. The incidence of amyloidosis ranges 0.3–8.0 : 100,000 [2]. ATTR is a systemic condition and can affect multiple organ systems including but not limited to the cardiac and nervous systems. ATTR has an insidious clinical presentation and is often overlooked and frequently diagnosed late. Our patient is a prime example. His CHF was believed to have developed from hypertension, hyperlipidemia, and diabetes mellitus. The combination of dyspnea, fatigue, weakness, and neuropathies in the form of bilateral carpel tunnel syndrome (CTS) and lumbar spinal stenosis (LSS) should rise a high suspicion for CA as exemplified in our case [3–5]. ATTRv can present with a spectrum of signs and symptoms depending on the genetic mutation with some presenting with more neurologic or cardiac manifestations [3, 6]. Val122Ile and TTR-V142I are commonly seen in AA with a prevalence of 3.4% and commonly presents with late-onset restrictive cardiomyopathies as seen in our patient [1, 3, 4, 6, 7].  Table 1 provides several findings that should prompt clinicians to pursue work-up for amyloidosis.

In contrast with AL amyloidosis where the amyloid protein is produced by malignant cells, the pathophysiology of both forms of ATTR is caused by unstable transthyretin tetramers produced by the liver that become misfolded leading to degradation into preamyloid monomers and eventual aggregation as amyloid fibrils [1, 2, 4]. A nonfibrillar glycoprotein called serum amyloid P complex (SAP) reversibly binds to amyloid fibrils making them insoluble and inhibiting their destruction [1, 2]. The amyloid fibrils begin to deposit into various tissues including the nerves and heart leading to clinical pathology. Deposition of the amyloid fibrils in the myocardium causes the chambers to stiffen leading to conduction disturbances and diastolic dysfunction.

Diagnosing CA remains a clinical challenge. Endomyocardial biopsy prevails as the gold standard and definitive diagnostic method of ATTR-CA with nearly 100% sensitivity and positivity. Tissue demonstrates apple-green birefringence under polarized light with Congo red stain [1, 3, 4, 8]. Afterwards, the tissue sample should undergo mass spectroscopy to evaluate for the specific subtype of ATTR. The invasive nature of the endomyocardial biopsy has stimulated alternative forms of diagnosis. Bone nuclear scintigraphy is currently the noninvasive diagnostic test of choice with 92% sensitivity and 95% specificity [1, 3, 8, 9]. The radiotracer technetium-pyrophosphate (^99m^Tc-PYP), technetium-99m 3,3-diphosphono-1,2-propanodicarboxylic acid (^99m^Tc-DPD) is used in Europe and is injected peripherally followed by imaging one hour and three hours later [4, 9, 10]. There are two different forms of interpretation. The first interpretation, a semiquantitative approach, compares the uptake of ^99m^Tc-PYP in the heart compared to the ribs. Hearts with uptake graded as no uptake, less than, equal to, or greater than the ribs are graded as 0, 1, 2, and 3, respectively [1, 8, 9]. Grades 2 and 3 are considered diagnostic for ATTR. The second interpretation compares circular regions of interest over the heart to the contralateral lung (H/CL ratio). A H/CL ratio ≥1.5 is considered diagnostic [1].

Appropriate diagnosis of CA is imperative for treatment and management. CHF arising from CA requires a different approach as it frequently does not respond to traditional CHF treatments [1, 3]. Beta blockers decompensate the patient via chronotropic insufficiency by decreasing the heart rate that was compensating for the poor stroke volume. Angiotensin-converting enzyme inhibitors and angiotensin receptor blockers lead to symptomatic hypotension and exacerbate underlying renal insufficiency. Nondihydropyridine calcium channel blockers can potentially bind to the amyloid fibrils resulting in heart block or shock. Digoxin precipitates arrhythmias leading to sudden cardiac death [1, 3].

In the last decade, CA went from untreatable to treatable. Tafamidis was the first drug approved for CA. Its mechanism of action works by binding to the thyroglobulin receptor on transthyretin and stabilizes defective transthyretin tetramers preventing degradation into insoluble monomers [4, 7, 11]. The ATTR-ACT trial was a randomized, phase III study of tafamidis versus placebo for the treatment of patients with ATTR that resulted in a decrease in all-cause mortality (29.5% vs. 42.9%) and cardiovascular-related hospitalizations (0.48 vs. 0.70 hospitalizations per year) for tafamidis versus placebo [12]. Diflunisal is a nonsteroidal anti-inflammatory drug that has been found to be a transthyretin stabilizer [1, 3, 11]. Similar to tafamidis, diflunisal binds to the thyroxine site on the transthyretin tetramer preventing destabilization and formation of amyloid fibrils [5]. Patisiran was developed as a small interfering RNA molecule that prevents the production of transthyretin at the chromosomal level [4, 11]. Inotersen is an antisense oligonucleotide responsible for inhibiting hepatic production of transthyretin [1, 3, 11].

In conclusion, amyloidosis is a medical condition with emerging clinical significance and may not be as rare of a condition as once believed. Due to its nonspecific symptoms, it has been underappreciated, underdiagnosed, and undertreated for decades. This clinical vignette hopes to increase awareness of the clinical impact of amyloidosis and the ability to be treated when promptly recognized. Therapeutic options continue to grow as will survival outcomes as the medical community gains more understanding and appreciation for this cryptic disease.

## Figures and Tables

**Table 1:** Red flags symptoms and findings of ATTR amyloidosis.

	Findings
Clinical symptoms	CHF without hypertension, bilateral CTS, LSS, newly diagnosed CHF over the age of 60, angina without CAD, peripheral neuropathy, dysautonomia, and repeated mild troponin increases [3, 5, 7]
Electrocardiogram	Diffuse low voltage, low voltage to mass ratio, evidence of pseudoinfarction, pathologic Q-waves without prior infarction or wall motion abnormality, and heart blocks [1]
Echocardiogram	Left ventricular wall thickness ≥12 mm, left atrial enlargement, and diastolic dysfunction. Amyloid appears speckled in the myocardium [1, 3, 7, 8].
Cardiac MRI	LGE progresses from the subendocardiocardial tissue to a more diffuse pattern as the amyloid infiltration increases [1]

CHF, congestive heart failure; CTS, carpel tunnel syndrome; LLS, lumbar spinal stenosis; CAD, coronary artery disease; LGE, late gadolinium enhancement.
